# Brain multiplexes reveal morphological connectional biomarkers fingerprinting late brain dementia states

**DOI:** 10.1038/s41598-018-21568-7

**Published:** 2018-03-07

**Authors:** Ines Mahjoub, Mohamed Ali Mahjoub, Islem Rekik, Alzheimer’s Disease Neuroimaging Initiative, Michael Weiner, Paul Aisen, Ronald Petersen, Cliford Jack, William Jagust, John Trojanowki, Arthur Toga, Laurel Beckett, Robert Green, Andrew Saykin, John Morris, Leslie Shaw, Jefrey Kaye, Joseph Quinn, Lisa Silbert, Betty Lind, Raina Carter, Sara Dolen, Lon Schneider, Sonia Pawluczyk, Mauricio Beccera, Liberty Teodoro, Bryan Spann, James Brewer, Helen Vanderswag, Adam Fleisher, Judith Heidebrink, Joanne Lord, Sara Mason, Colleen Albers, David Knopman, Kris Johnson, Rachelle Doody, Javier Villanueva-Meyer, Munir Chowdhury, Susan Rountree, Mimi Dang, Yaakov Stern, Lawrence Honig, Karen Bell, Beau Ances, Maria Carroll, Mary Creech, Erin Franklin, Mark Mintun, Stacy Schneider, Angela Oliver, Daniel Marson, Randall Grifth, David Clark, David Geldmacher, John Brockington, Erik Roberson, Marissa Natelson Love, Hillel Grossman, Efe Mitsis, Raj Shah, Leyla deToledo-Morrell, Ranjan Duara, Daniel Varon, Maria Greig, Peggy Roberts, Marilyn Albert, Chiadi Onyike, Daniel D’Agostino, Stephanie Kielb, James Galvin, Brittany Cerbone, Christina Michel, Dana Pogorelec, Henry Rusinek, Mony de Leon, Lidia Glodzik, Susan De Santi, P. Doraiswamy, Jefrey Petrella, Salvador Borges-Neto, Terence Wong, Edward Coleman, Charles Smith, Greg Jicha, Peter Hardy, Partha Sinha, Elizabeth Oates, Gary Conrad, Anton Porsteinsson, Bonnie Goldstein, Kim Martin, Kelly Makino, M. Ismail, Connie Brand, Ruth Mulnard, Gaby Thai, Catherine Mc-Adams-Ortiz, Kyle Womack, Dana Mathews, Mary Quiceno, Allan Levey, James Lah, Janet Cellar, Jefrey Burns, Russell Swerdlow, William Brooks, Liana Apostolova, Kathleen Tingus, Ellen Woo, Daniel Silverman, Po Lu, George Bartzokis, Neill Graf-Radford, Francine Parftt, Tracy Kendall, Heather Johnson, Martin Farlow, Ann Marie Hake, Brandy Matthews, Jared Brosch, Scott Herring, Cynthia Hunt, Christopher Dyck, Richard Carson, Martha MacAvoy, Pradeep Varma, Howard Chertkow, Howard Bergman, Chris Hosein, Sandra Black, Bojana Stefanovic, Curtis Caldwell, Ging-Yuek Robin Hsiung, Howard Feldman, Benita Mudge, Michele Assaly, Elizabeth Finger, Stephen Pasternack, Irina Rachisky, Dick Trost, Andrew Kertesz, Charles Bernick, Donna Munic, Marek-Marsel Mesulam, Kristine Lipowski, Sandra Weintraub, Borna Bonakdarpour, Diana Kerwin, Chuang-Kuo Wu, Nancy Johnson, Carl Sadowsky, Teresa Villena, Raymond Scott Turner, Kathleen Johnson, Brigid Reynolds, Reisa Sperling, Keith Johnson, Gad Marshall, Jerome Yesavage, Joy Taylor, Barton Lane, Allyson Rosen, Jared Tinklenberg, Marwan Sabbagh, Christine Belden, Sandra Jacobson, Sherye Sirrel, Neil Kowall, Ronald Killiany, Andrew Budson, Alexander Norbash, Patricia Lynn Johnson, Thomas Obisesan, Saba Wolday, Joanne Allard, Alan Lerner, Paula Ogrocki, Curtis Tatsuoka, Parianne Fatica, Evan Fletcher, Pauline Maillard, John Olichney, Charles DeCarli, Owen Carmichael, Smita Kittur, Michael Borrie, T-Y Lee, Rob Bartha, Sterling Johnson, Sanjay Asthana, Cynthia Carlsson, Steven Potkin, Adrian Preda, Dana Nguyen, Pierre Tariot, Anna Burke, Nadira Trncic, Stephanie Reeder, Vernice Bates, Horacio Capote, Michelle Rainka, Douglas Scharre, Maria Kataki, Anahita Adeli, Earl Zimmerman, Dzintra Celmins, Alice Brown, Godfrey Pearlson, Karen Blank, Karen Anderson, Laura Flashman, Marc Seltzer, Mary Hynes, Robert Santulli, Kaycee Sink, Leslie Gordineer, Jef Williamson, Pradeep Garg, Franklin Watkins, Brian Ott, Henry Querfurth, Geofrey Tremont, Stephen Salloway, Paul Malloy, Stephen Correia, Howard Rosen, Bruce Miller, David Perry, Jacobo Mintzer, Kenneth Spicer, David Bachman, Nunzio Pomara, Raymundo Hernando, Antero Sarrael, Norman Relkin, Gloria Chaing, Michael Lin, Lisa Ravdin, Amanda Smith, Balebail Ashok Raj, Kristin Fargher

**Affiliations:** 1 BASIRA lab, CVIP group, School of Science and Engineering, Computing, University of Dundee, Dundee, UK; 2 LATIS lab, ENISo – National Engineering School of Sousse, Sousse, Tunisia; 3 Magnetic Resonance Unit at the VA Medical Center and Radiology, Medicine, Psychiatry and Neurology, University of California, San Francisco, USA; 4 San Diego School of Medicine, University of California, California, USA; 5 Mayo Clinic, Rochester, Minnesota USA; 6 Mayo Clinic, Rochester, USA; 7 University of California, Berkeley, USA; 8 University of Pennsylvania, Pennsylvania, USA; 9 University of Southern California, California, USA; 10 University of California, Davis, California, USA; 11 MPH Brigham and Women’s Hospital/Harvard Medical School, Massachusetts, USA; 12 Indiana University, Indiana, USA; 13 Washington University St. Louis, St Louis, Missouri USA; 14 Oregon Health and Science University, Oregon, USA; 15 University of California–San Diego, California, USA; 16 University of Michigan, Ann Arbor, Michigan USA; 17 Baylor College of Medicine, Houston, State of Texas USA; 18 Columbia University Medical Center, New York, South Carolina USA; 19 University of Alabama, Birmingham, Alabama USA; 20 Mount Sinai School of Medicine, New York, USA; 21 Rush University Medical Center, Rush University, Chicago, Illinois USA; 22 Wien Center, Miami, Florida USA; 23 Johns Hopkins University, Baltimore, Maryland USA; 24 New York University, New York, NY USA; 25 Duke University Medical Center, Durham, North Carolina USA; 26 University of Kentucky, Lexington, Kentucky USA; 27 University of Rochester Medical Center, Rochester, NY USA; 28 University of California, Irvine, California, USA; 29 University of Texas Southwestern Medical School, Dallas, Texas USA; 30 Emory University, Atlanta, Georgia USA; 31 University of Kansas, Medical Center, Kansas, USA; 32 University of California, Los Angeles, California, USA; 33 Mayo Clinic, Jacksonville, Jacksonville, USA; 34 Yale University School of Medicine, New Haven, Connecticut USA; 35 McGill University, Montreal-Jewish General Hospital, Montreal, Canada; 36 Sunnybrook Health Sciences, Ontario, USA; 37 U.B.C. Clinic for AD & Related Disorders, Vancouver, BC Canada; 38 Cognitive Neurology - St. Joseph’s, Ontario, USA; 39 Cleveland Clinic Lou Ruvo Center for Brain Health, Ohio, USA; 40 Northwestern University, San Francisco, USA; 41 Premiere Research Inst (Palm Beach Neurology), west Palm Beach, USA; 42 Georgetown University Medical Center, Washington, DC USA; 43 Brigham and Women’s Hospital, Massachusetts, USA; 44 Stanford University, California, USA; 45 Banner Sun Health Research Institute, Sun City, AZ 85351 USA; 46 Boston University, Massachusetts, USA; 47 Howard University, Washington, DC USA; 48 Case Western Reserve University, Ohio, USA; 49 University of California, Davis – Sacramento, California, USA; 50 Neurological Care of CNY, Liverpool, NY 13088 USA; 51 Parkwood Hospital, Pennsylvania, USA; 52 University of Wisconsin, Wisconsin, USA; 53 University of California, Irvine, BIC USA; 54 Banner Alzheimer’s Institute, Phoenix, AZ 85006 USA; 55 Dent Neurologic Institute, Amherst, NY USA; 56 Ohio State University, Ohio, USA; 57 Albany Medical College, Albany, NY USA; 58 Hartford Hospital, Olin Neuropsychiatry Research Center, Hartford, Connecticut USA; 59 Dartmouth-Hitchcock Medical Center, Lebanon, New Hampshire USA; 60 Wake Forest University Health Sciences, Winston-Salem, North Carolina USA; 61 Rhode Island Hospital, state of Rhode Island, Providence, RI 02903 USA; 62 Butler Hospital, Providence, Rhode Island USA; 63 University of California, San Francisco, USA; 64 Medical University South Carolina, Charleston, SC 29425 USA; 65 Nathan Kline Institute, Orangeburg, New York, USA; 66 Cornell University, Ithaca, New York, USA; 67 USF Health Byrd Alzheimer’s Institute, University of South Florida, Tampa, FL 33613 USA

## Abstract

Accurate diagnosis of mild cognitive impairment (MCI) before conversion to Alzheimer’s disease (AD) is invaluable for patient treatment. Many works showed that MCI and AD affect functional and structural connections between brain regions as well as the shape of cortical regions. However, ‘shape connections’ between brain regions are rarely investigated -e.g., how morphological attributes such as cortical thickness and sulcal depth of a specific brain region change in relation to morphological attributes in other regions. To fill this gap, we unprecedentedly design morphological brain multiplexes for late MCI/AD classification. Specifically, we use structural T1-w MRI to define morphological brain networks, each quantifying similarity in morphology between different cortical regions for a specific cortical attribute. Then, we define a brain multiplex where each intra-layer represents the morphological connectivity network of a specific cortical attribute, and each inter-layer encodes the similarity between two consecutive intra-layers. A significant performance gain is achieved when using the multiplex architecture in comparison to other conventional network analysis architectures. We also leverage this architecture to discover morphological connectional biomarkers fingerprinting the difference between late MCI and AD stages, which included the right entorhinal cortex and right caudal middle frontal gyrus.

## Introduction

Alzheimer Disease (AD) is one of the most devastating neurodegenerative diseases, affecting memory as well as cognitive functions of the human brain. With the absence of immediate treatment for patients diagnosed with AD, an accurate diagnosis of AD in an earlier stage propels early clinical interventions that could help slow down irreversible cognitive decline. Specifically, an intermediate stage exists between AD and normal control (NC) which is Mild Cognitive Impairment (MCI), where unlike AD, the memory deficits in MCI patients may remain relatively stable for years. The MCI stage is regarded as very critical as patients can still benefit from adequate clinical interventions before conversion to AD.

Considering the increasing number of brain imaging datasets on dementia and particularly AD, several methods based on neuroimaging processing and machine-learning have been developed in the purpose of early detection of AD conversion at the MCI stage. Hence, detecting brain biomarkers in the stage of MCI may allow the individualization of effective treatment to demented patients. Remarkably, the majority of these methods have extensively relied on resting state functional and diffusion magnetic resonance imaging (MRI)^1–8^. Some works proposed brain network analysis methods using noninvasive diffusion MRI for AD diagnosis, where structural connectivities were measured using the degree of white matter connectivity between the associated pairs of ROIs^4,8^. On the other hand, several studies used functional brain networks which mostly focused on characterizing the pairwise correlation (e.g., Pearson Correlation) between ROIs. Recently, more advanced studies proposed novel functional connectivity (FC) representations to model brain networks at different connectional levels. For example, Yu *et al*. proposed a novel method to construct brain FC by taking advantage of both Pearson Correlation and sparse learning^1^. While some works only used high-order FC^3^ considering the relationships between pairs of ROIs, more recent studies integrated both low-order and high-order FC networks along with interactions between the two levels^7^. However, analysis of functional networks is typically limited by the choice of a single or multiple thresholds for examining network topology, which may discard many important and discriminative brain connectivities. Moreover, while functional MRI can produce spurious and noisy connectomes, diffusion MRI can produce biased and largely variable structural connectomes depending on the employed fiber tractography method^9^. Besides, both structural and functional modalities are rarely acquired in a conventional clinical routine. Additionally, distinguishing between late MCI (LMCI) patients, who might be on the verge to convert to AD, and AD patients is a much more challenging classification task than that of AD vs. NC or early MCI (EMCI) vs. AD. Due to the very subtle brain changes between LMCI and AD brain changes, LMCI/AD classification task remains a hard problem to solve, that has been hardly addressed in the AD literature^10,11^.

On the other hand, many other studies have demonstrated the importance of considering cortical measures derived from the multi-folded surface of the cerebral cortex for AD diagnosis, such as the cortical thickness^12–15^. Specifically, cortical thickness is considered as a biomarker of AD progression, which provides insight into normal brain development and neurodegenerative disorders since it is correlated with changes in cognitive performance^15–18^. For instance, Frisoni *et al*. showed reduction in cortical thickness in AD subjects compared with control subjects^15^. Thus, many voxel-based methods^16,17^ or region-based methods^18^ heavily relied on morphological features, including volumetric cortical thickness measurements from MRI, for AD diagnosis. However, all these methods were based on volumetric cortical thickness analysis, while there is evidence that AD alters not only volume-based cortical measures, but also the shape of cortical regions -e.g., cortical thinning at different levels^19^. For this reason, other studies explored cortical thickness using surface-based methods involving spectral shape description^20^, or combining shape-derived features with voxel features^21,22^. However, these approaches considered the morphological features at only the vertex-level. To the best of our knowledge, none of existing network-based analysis methods for disentangling late AD states investigated the *morphological* connectivities between ROIs using structural T1-w MRI-i.e., modeling how the morphology of different brain regions may be affected in relation to one another. Moreover, since AD may affect the complex relationships between a set of attributes in different cortical regions, one cannot rely on a single cortical attribute to examine how the brain is progressively altered by different stages of AD. A more comprehensive approach would consider multiple cortical attributes (e.g., sulcal depth, cortical thickness), each of these representing a single view of cortex morphology to quantify brain morphology. In this paper, we propose the first multi-view morphological brain connectivity using four different cortical attributes: cortical thickness network, sulcal depth network, average curvature network, and principal curvature network. Then, based on this multi-view connectional representation of brain morphology, we further propose novel network architecture that would allow us to investigate the complex relationship between these views for identifying late MCI morphological connectional biomarkers distinguishing between LMCI and AD.

Typically, the majority of network-based methods developed for MCI/AD classification diagnosis overlooked the high-order relationship between different brain connectional layers. A few recent network-analysis works proposed for classification tasks between different AD stages (e.g., early MCI, late MCI), used one-layer network representation^23^, a multi-layer (i.e. set of concatenated networks) network^24^ or high-order networks^10,11^. Specifically, the recently proposed high-order functional connectivity networks for MCI/AD diagnosis^23^ integrated new high-level features that encode how different brain region pairs, instead of two brain regions, functionally interact with each other. Nevertheless, it will be possible to further consider other new connections through exploring how different network pairs interact with each another (and not only brain region pairs). This nicely led us to the concept of a *multiplex network*, which was historically coined to indicate the presence of more than one relationship between the same actors of a social network^25^. Some previous methods^26–30^ have explored multiplexes to study brain networks (e.g., structural, functional). These multiplex networks (or multiplexes) allow multiple types of relationships to be represented in modelling brain connectivities, thus capturing higher levels of complexity between brain regions. However, all the mentioned studies investigated multiplex as a multi-layer network without exploring similarity networks that encode the relationship between consecutive brain connectivity layers. For instance, Battiston *et al*. used multiplexes as a two-layer network (functional and anatomical) to extract brain subgraphs while overlooking the inter-layer that perform high-order connectivities^27^. Moreover, these approaches either relied on fMRI, combine fMRI with structural MRI, or used different modalities such as MRI with PET^26^; but none explored morphological brain network each based on a specific attribute of the cortical surface, with the notable exception of recent works^31,32^ targeting early dementia and autism spectrum disorder diagnosis.

To address this limitation, we further propose a morphological brain multiplex interleaving a set of two different layers: an intra-layer which represents the morphological connectivity network of a specific cortical attribute, and an inter-layer (or a similarity layer) which computes the Pearson Correlation between two consecutive intra-layers. The proposed architecture leverages both morphological networks and the correlational relationship between each two consecutive layers. However, different similarity networks can be extracted by varying the order of layers. Hence, we define an ensemble of morphological brain multiplexes, each capturing complex network-to-network relationships for predefined set of cortical attributes by reordering at each time different intra-layers, with the exception of the first intra-layer, to capture new similarity networks. We aim by this architecture to discover morphological connectional biomarkers distinguishing between AD and LMCI patients, which can be clinically useful for early detection of AD conversion at MCI stage.

## Results

### Data processing and parameters

In our study, we used 77 subjects (41 AD and 36 LMCI) from ADNI GO public dataset, each with structural T1-w MR image^33^. Data used in the preparation of this article were obtained from the Alzheimer’s Disease Neuroimaging Initiative (ADNI) database (adni.loni.usc.edu). The ADNI was launched in 2003 as a public-private partnership, led by Principal Investigator Michael W. Weiner, MD. The primary goal of ADNI has been to test whether serial magnetic resonance imaging (MRI), positron emission tomography (PET), other biological markers, and clinical and neuropsychological assessment can be combined to measure the progression of mild cognitive impairment (MCI) and early Alzheimer’s disease (AD). We used FreeSurfer processing pipeline^34^ to reconstruct both right (RH) and left (LH) cortical hemispheres for each subject from T1-w MRI^35^. Then we parcellated each cortical hemisphere into 35 cortical regions using Desikan-Killiany cortical atlas^35^. Using FreeSurfer pipeline, each vertex on the cortical surface was assigned four cortical attributes: maximum principal curvature, cortical thickness, sulcal depth, and average curvature.

For the deep similarity network architecture, we used two levels (*l* = 0, *l* = 1). We defined *K* = 6 multiplexes using 4 cortical attributes, where multiplex ${ {\mathcal M} }_{1}$ includes morphological networks generated using different cortical attributes {*N*_1_, *N*_2_, *N*_3_, *N*_4_}, ${ {\mathcal M} }_{2}$ includes {*N*_1_, *N*_2_, *N*_4_, *N*_3_}, ${ {\mathcal M} }_{3}$ includes {*N*_1_, *N*_3_, *N*_4_, *N*_2_},$\,{ {\mathcal M} }_{4}$ includes {*N*_1_, *N*_3_, *N*_2_, *N*_4_},$\,{ {\mathcal M} }_{5}$ includes {*N*_1_, *N*_4_, *N*_2_, *N*_3_}, and ${ {\mathcal M} }_{6}$ includes {*N*_1_, *N*_4_, *N*_3_, *N*_2_}. For each cortical region, *N*_1_ denotes the mean maximum principal curvature, *N*_2_ denotes the mean cortical thickness, *N*_3_ denotes the mean sulcal depth and *N*_4_ denotes the mean of average curvature.

### Data distribution

Table 1 displays the gender/age distribution for both AD and LMCI groups. Both groups were matched in gender and age.

**Table 1:** Data distribution. M: male. F: female. Total: total number of subjects in each group. Std: standard deviation.

	AD	LMCI
M	23	20
F	18	16
Total	41	36
Mean age	75.27	72.54
Std age	8.72	6.10

### Comparison methods

We compared our proposed architectures with two conventional methods: (1) one-layer network architecture, and (2) concatenated multi-layer network architecture. For the first baseline method, we used the designed *M* morphological brain networks. For the second baseline method, we constructed the multi-layer network through concatenating all morphological networks in a large network ${\mathscr{N}}=\{{N}_{1},\mathrm{..},{N}_{M}\}$ of size *R* × *R* × *M* (R = 35, M = 4).

### Evaluation

**Figure 1:**
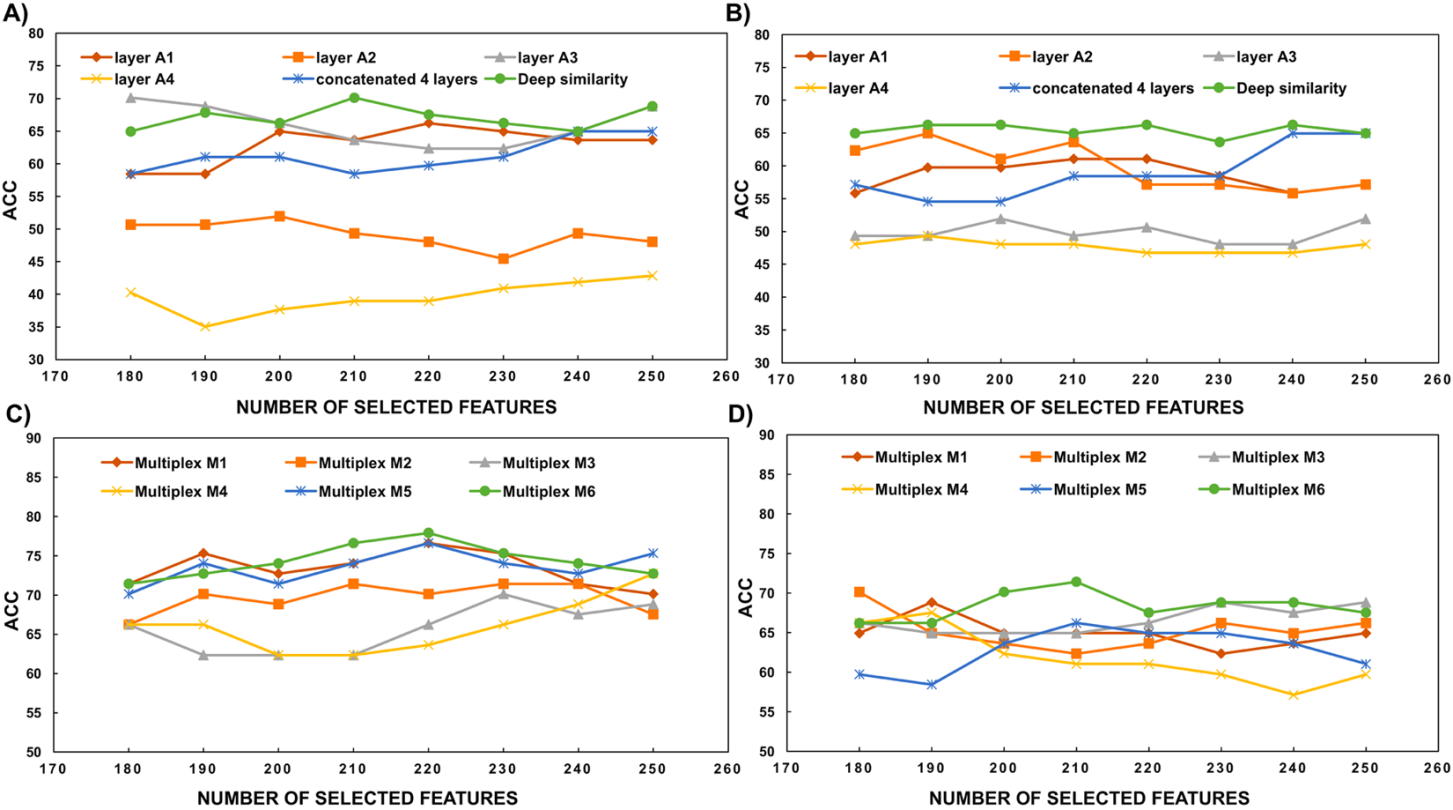
Influence of the selected features number on the accuracy of both baseline and proposed methods. (**A**) and (**B**) plot the accuracy curves against the number of selected features for deep similarity network compared with baseline methods for RH and LH, respectively. (**C**) and (**D**) plot the accuracy curves against the number of selected features for the 6 proposed multiplexes of right and left hemispheres respectively.

We evaluated our framework through varying the number of *K*_*f*_ selected features from 180 to 250 by adding 10 features at each evaluation step (Fig. 1). We noted that for the majority of the selected features’ dimensions, the deep similarity architecture increased the classification performance in comparison with the conventional methods (i.e., one-layer network and concatenated 4-layer network), for both left and right hemispheres. Remarkably, the classification accuracies highly improved when using particular brain multiplexes. Table 2 displays the average classification accuracies for baseline methods as well as for the proposed architectures. The best average accuracy was achieved by multiplex 6 (respectively including {*N*_1_, *N*_4_, *N*_3_, *N*_2_} cortical attributes as intra-layers) for LH (68.61%), while it was achieved by multiplex 5 (respectively including {*N*_1_, *N*_4_, *N*_2_, *N*_3_} cortical attributes as intra-layers) for RH (72.25%).

**Table 2:** LMCI/AD average classification accuracy for the proposed morphological network architectures by varying the number of top selected features from 180 to 250, with an incremental step of 10 features. ACC: accuracy. SEN: sensitivity. SPE: specificity.

	ACC		SEN		SPE	
	LH	RH	LH	RH	LH	RH
Layer *A*_1_	57.69	61.15	56.59	57.73	58.53	67.37
Layer *A*_2_	59.74	51.35	55.21	41.91	66.76	50.55
Layer *A*_3_	49.43	66.47	47.22	63.39	51.22	66.46
Layer *A*_4_	47.18	40.34	43.40	41.27	50.30	41.86
*A*_1_ − *A*_4_	59.03	61.51	55.90	59.02	61.89	63.10
Deep similarity	65.32	64.84	67.70	68.05	63.71	66.15
Multiplex ${ {\mathcal M} }_{1}$	65.06	71.42	66.32	**75.00**	65.54	71.95
Multiplex ${ {\mathcal M} }_{2}$	65.70	68.47	68.40	66.66	64.02	72.25
Multiplex ${ {\mathcal M} }_{3}$	66.38	66.58	69.09	61.68	65.24	69.20
Multiplex ${ {\mathcal M} }_{4}$	63.61	66.47	65.97	67.36	62.49	64.93
Multiplex ${ {\mathcal M} }_{5}$	61.93	**72.25**	56.25	72.56	**66.76**	**74.39**
Multiplex ${ {\mathcal M} }_{6}$	**68.61**	70.95	**73.61**	74.65	65.45	74.08

**Figure 2:**
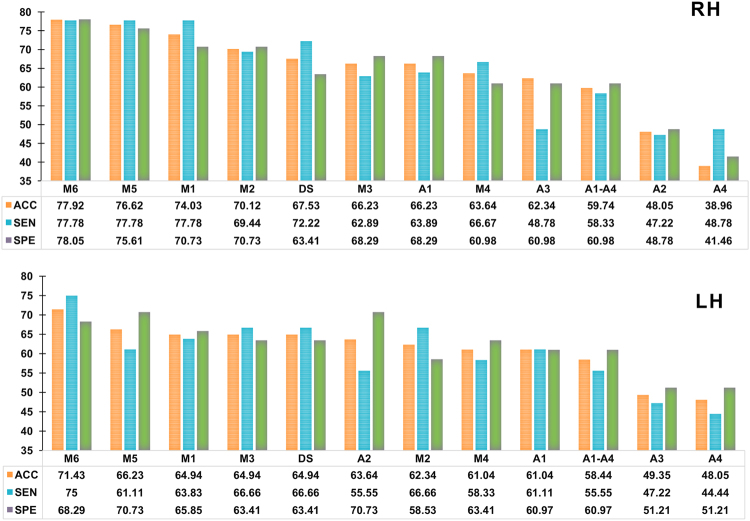
Best performances reached respectively by right hemisphere RH and left hemisphere LH, using different architecture networks. ‘A’ denotes cortical attribute, ‘DS’ denotes deep similarity, and ‘M’ denotes Multiplex.

Notably, the best accuracies were obtained using a number of features equal to 220 and 210 for RH and LH, respectively (Fig. 2). When using one-layer morphological networks, the classification accuracy only reached 63.64% for LH and 66.23% for RH. These classification results decreased when concatenating features extracted from all morphological networks (*level 0*), as the classification rate was limited to 59.74% for RH and 58.44% for LH. However, when we further integrated the similarity networks between all pairs of layers in the proposed deep similarity network architecture (*level 1*), the performance significantly increased for both RH (67.53%) and LH (64.94%) compared to one-layer network and concatenated one-layer networks architectures. For both hemispheres, the highest accuracies were achieved using multiplex ${ {\mathcal M} }_{6}$, multiplex ${ {\mathcal M} }_{5}$, and multiplex ${ {\mathcal M} }_{1}$. Specifically, multiplex ${ {\mathcal M} }_{6}$ achieved the best accuracy among all architecture networks with a classification accuracy peaking at 77.92% for RH and 71.43% for LH. This shows that the proposed similarity networks allow to better discriminate between LMCI and AD subjects. This was also reflected by the percentages of discriminative features belonging to the similarity inter-layer networks for each multiplex as shown in Table 3. We note that for some multiplexes, more than 50% of *K*_*f*_ discriminative features lied in the network *inter-layers* -i.e., similarity networks.

**Table 3:** Percentage of discriminative features belonging to the similarity inter-layers and the intra-layers for each of the proposed multiplexes.

	% of intra-layers		% of inter-layers	
	LH	RH	LH	RH
Multiplex ${ {\mathcal M} }_{1}$	54.12	59.44	45.88	40.56
Multiplex ${ {\mathcal M} }_{2}$	50.27	35.52	**49.73**	**64.74**
Multiplex ${ {\mathcal M} }_{3}$	43.66	39.59	**56.34**	**60.41**
Multiplex ${ {\mathcal M} }_{4}$	38.15	51.75	**61.85**	**48.24**
Multiplex ${ {\mathcal M} }_{5}$	42.00	56.00	**58.00**	44.00
Multiplex ${ {\mathcal M} }_{6}$	53.54	62.77	46.45	37.23

### Identified morphological connectional biomarkers for LMCI/AD classification

We further explored our multiplex architecture and morphological networks to identify morphological connectional biomarkers that discriminate between LMCI and AD patients. Since we aimed to find the most discriminative morphological connections, we chose the brain multiplex with the highest discriminative power. For LH, we found that the discriminative power of multiplex ${ {\mathcal M} }_{6}$ was the most reproducible since it gave the best average accuracy across different numbers of selected features as well as the best accuracy (for *K*_*f*_ = 210) in comparison with all other network architectures. As for RH, multiplex ${ {\mathcal M} }_{5}$ achieved the best mean average across different numbers of selected features, while we noted the highest accuracy reached by multiplex ${ {\mathcal M} }_{6}$ for a number of features equal to 220. Hence, we selected multiplex ${ {\mathcal M} }_{6}$ achieving the best accuracies for both hemispheres to discover morphological connectional biomarkers, using the specific number of discriminative features 220 (77.92%) and 210 (71.43%) for RH and LH, respectively.

**Figure 3:**
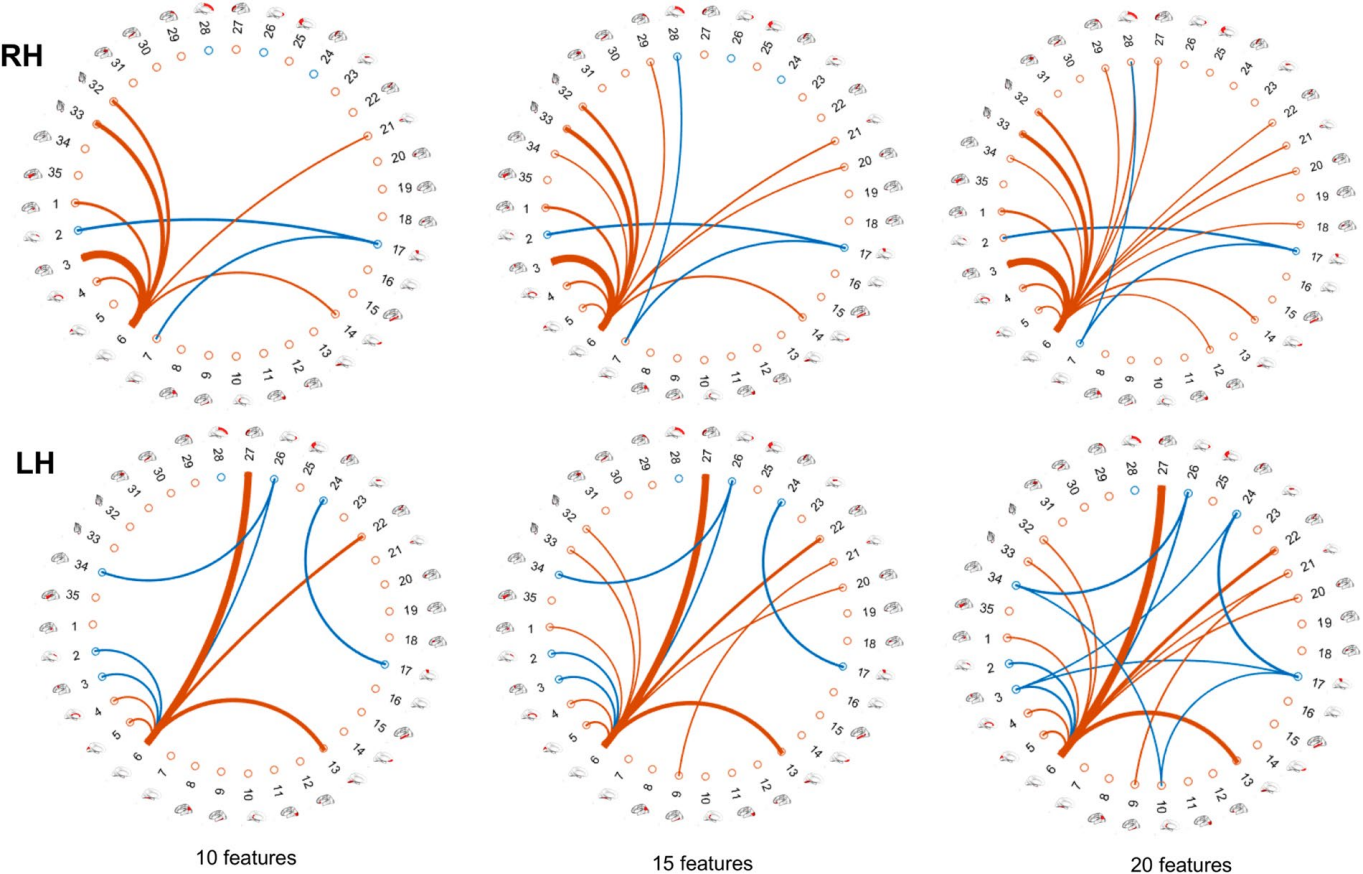
Most discriminative morphological cortical network connections between LMCI and AD for RH and LH, respectively.

In Fig. 3, we visualized using circular graphs the top most frequently selected morphological brain connectivities in multiplex ${ {\mathcal M} }_{6}$. Circular graphs were plotted for the top 10, 15 and 20 discriminative features, respectively. The thickness of each edge connecting a pair of ROIs represents the normalized rank of the discriminative brain connection. The most discriminative connections with the highest normalized ranks have thick edges, while those with less discriminative power have thinner edges. Blue edges denote connections belonging to a multiplex inter-layer, while red edges denote connections falling into a multiplex intra-layer.

We noted that 20% (resp. 50%) of the top 10 discriminative features were located in the multiplex inter-layers for RH (resp. LH). Using the normalized ranks, the most discriminative connectional features for RH connected the Entorhinal Cortex (EC) (region 6) and the Caudal Middle Frontal Gyrus (CMFG) (region 3), EC and Temporal Pole (TP) (region 33), EC and Frontal Pole (FP) (region 32), EC and Bank of the Superior Temporal Sulcus (BSTS) (region 1) and the fifth connectivity was between the Paracentral Lobule (PL) (region 17) and Caudal Anterior-cingulate Cortex (CAC) (region 2), respectively. As for the LH, the most discriminative features connected the EC and the Rostral Middle Frontal Gyrus (RMFG) (region 27), EC and Lingual Gyrus (LG) (region 13), EC and Postcentral Gyrus (region 22), EC and CAC, and PL and Precentral Gyrus (region 24), respectively. We noted that the most discriminative morphological hub node in multiplex ${ {\mathcal M} }_{6}$ for both hemispheres was the entorhinal cortex, where the top four discriminative connections with the highest normalized ranks branched from it.

Besides, even when we increased the number of discriminative features from 10 to 20, new connectivities appeared, most of them emerged from the EC. Moreover, the percentage of the top discriminative features belonging to inter-layers was low for RH (~15%) compared to LH (~45%). We also note that the majority of the most discriminative morphological brain connections fell into the 5^*th*^ layer (*A*_3_), which represents the mean sulcal depth attribute (Tables 4 and 5).

**Table 4:** Top 10 discriminative morphological connections in the cortex with their corresponding layers for the right hemisphere.

Rank	First region	Second region	Layer
1	Entorhinal Cortex	Caudal Middle Frontal Gyrus	Mean sulcal depth
2	Temporal Pole	Entorhinal Cortex	Mean sulcal depth
3	Frontal Pole	Entorhinal Cortex	Mean sulcal depth
4	Entorhinal Cortex	Bank of the superior Temporal Sulcus	Mean sulcal depth
5	Paracentral Lobule	Caudal interior cingulate cortex	Similarity between mean sulcal depth and mean maximum principal curvature
6	Entorhinal Cortex	Unmeasured Corpus Callosum	Mean sulcal depth
7	Medial Orbital Frontal Cortex	Entorhinal Cortex	Mean sulcal depth
8	Paracentral Lobule	Fusiform Gyrus	Mean sulcal depth
9	Pericalcarine Cortex	Entorhinal Cortex	Mean sulcal depth
10	Superior Frontal Gyrus	Entorhinal Cortex	Mean sulcal depth

**Table 5:** Top 10 discriminative morphological connections in the cortex with their corresponding layers for the left hemisphere.

Rank	First region	Second region	Layer
1	Rostral Middle Frontal Gyrus	Entorhinal Cortex	Mean sulcal depth
2	Lingual Gyrus	Entorhinal Cortex	Mean sulcal depth
3	Postcentral Gyrus	Entorhinal Cortex	Mean sulcal depth
4	Entorhinal Cortex	Caudal Anterior-Cingulate Cortex	Similarity between mean maximum principal curvature and mean of average curvature
5	Precentral Gyrus	Paracentral Lobule	Similarity between mean of average curvature and mean sulcal depth
6	Transverse Temporal Cortex	Rostral Anterior Cingulate Cortex	Similarity between mean of average curvature and mean sulcal depth
7	Entorhinal Cortex	Cuneus Cortex	Mean sulcal depth
8	Entorhinal Cortex	Caudal Middle Frontal Gyrus	Similarity between mean maximum principal curvature and mean of average curvature
9	Rostral Anterior Cingulate Cortex	Entorhinal Cortex	Similarity between mean maximum principal curvature and mean of average curvature
10	Entorhinal Cortex	Unmeasured Corpus Callosum	Mean sulcal depth

More importantly, while most discriminative features belonging to intra-layers emerged from EC for both left and right hemispheres, those belonging to inter-layers emerged from the fusiform gyrus and the paracentral lobule for RH. The same regions were present in LH with new other hub nodes including the precentral gyrus, transverse temporal cortex, rostral anterior cingulate cortex, and isthmus-cingulate cortex.

## Discussion

We proposed a novel representation of brain connectivity to identify connectional biomarkers based on the *morphology* of the cerebral cortex for distinguishing between late mild cognitively impaired patients and Alzheimer’s disease patients. In this study, we unprecedentedly investigated the role of several morphological connectivity networks as well as the correlation between them to discover morphological connectional brain biomarker fingerprinting the difference between LMCI and AD states. In particular, we proposed two brain architectures: the deep similarity network and the multiplex network. While in the first architecture we simply concatenated all possible similarity networks with the main morphological network, in the second one we constructed similarity networks only between successive layers, and generated different multiplexes by reordering the morphological layers.

Our proposed architectures achieved better performances than one-layer morphological network and concatenated 4-layer networks. This shows that the aggregation of different similarities between morphological brain connections helps better discriminate between AD and LMCI patients compared to using a single morphological network or even all morphological networks without exploring their relationships (Fig. 2). Moreover, our multiplex architecture achieved the highest accuracies, which indicates that the similarity inter-layers between morphological networks are able to capture a higher-level discriminative information. This demonstrates that disease-driven changes in the cortical shape quantified using a specific cortical attribute can be also influenced by shape changes measured using a different cortical attribute.

Through using different cortical attributes and identifying the most of discriminative features by multiplex ${ {\mathcal M} }_{6}$, we found that the mean sulcal depth has the highest discriminative power (Tables 4 and 5). Sulcal depth has been identified in the literature as one of the quantitative measures of cerebral cortex, representing an important morphological biomarker for AD^36,37^. Im *et al*. presented a surface-based method that investigated changes of sulcal shape in MCI and AD, using sulcal depth and average mean curvature^36^. They showed that the progression of disease from NC to MCI and MCI to AD was coupled with shallowness in sulcal depth. The same finding^37^ was replicated by Yun *et al*., which proposed an automated sulcal depth measurement on cortical surface and highlighted that mean sulcal depth in MCI was lower than in NC.

The most discriminative morphological connectivities with the highest normalized ranks were established between EC and CMFG for RH, and EC and RMFG for LH. Many studies highlighted that RMFG is a discriminative region in AD diagnosis as well as CMFG^38^. It was also noted that about 18% of the CMFG atrophies in AD patients^38^.

One of the major findings of our study is the detection of morphological brain connectional biomarkers fingerprinting the distinction between LMCI and AD dementia brain states. We found that 85% (resp. 65%) of most RH (resp. LH) discriminative regions connected to the EC fingerprint LMCI/AD classification (Fig. 3). The EC has a major role in working memory processing^39–43^. Its importance was revealed due to its anatomical interconnection with the hippocampus, which is the major region responsible of memory formation^43,44^. EC role consists of generating coding schemes for new memories and storing them temporarily. It has numerous reciprocal connections with the hippocampus, specifically an effective connectivity in the hippocampus strongly depends on the connectivity among EC layers^45^.

Our findings based on cortical morphological connectivity were in line with previous studies, since the EC has been considered as a good biomarker for AD and MCI in the literature^46–50^. It has a great potential for detecting early memory decline and is considered as the region of early neurodegeneration caused by dementia. Velayudhan *et al*. examined the relationship between EC thickness, hippocampal volume and the whole brain volume, and showed that AD patients have thinner EC thickness and smaller hippocampal volume compared with MCI subjects^46^. The same hypothesis about the role of EC was demonstrated by the work of Thaker *et al*.^47^, which considered EC thickness as a marker of medial temporal and neocortical AD neuropathology. The review paper^48^ also highlighted the early EC atrophy detection as an important anatomical marker for MCI and AD, since it was remarkably highly correlated with the early pathological changes in AD. Besides, greater changes in the right EC were present compared with the left one, which substantiates our results, since we achieved the best multiplex-based LMCI/AD classification performance using the right hemisphere.

Our study has a few limitations. *First*, although we used different types of morphological attributes, we simply concatenated all derived connectivities to extract features without creating fused predictors of disease diagnosis. *Second*, though we identified key morphological connectional biomarkers for LMCI stage, mainly involving the entorhinal cortex, we did not investigate the connection of the discovered cortical regions to other non-cortical regions (e.g., EC to hippocampus). It is still not clear how the shape-based morphological connectivities of EC can be altered with the hippocampus connectivities such as functional or structural. *Third*, since MCI is a progressive disease, tracking the discriminative power of the identified morphological biomarkers can help better understand how the morphology of a specific discriminative region (e.g., EC thickness) gets altered progressively with time in cognition in relation to other cortical attributes^42,44^. *Fourth*, although the structural underpinning of morphological networks remains unclear, the co-vary brain regions were suggested as a result of mutually trophic influences or common experience related plasticity^51,52^. In particular, it was noted that the pattern of cortical thickness correlation of certain brain regions is similar to the underlying fiber connections from DTI tractography^53^. Gong *et al*. also pointed out that approximately 35–40% convergent connections exist between brain networks using thickness and diffusion measurements, which suggests that thickness correlations include exclusive information^54^.

In our future work, we will investigate longitudinal morphological connectivities to improve our framework as well as longitudinal morphological changes in the EC. Besides, we will use advanced methods for different morphological and similarity networks fusion^55^ while integrating other multimodal brain networks (e.g., resting-state functional networks and structural diffusion networks) into our proposed brain multiplex architectures^26^.

## Methods

**Figure 4:**
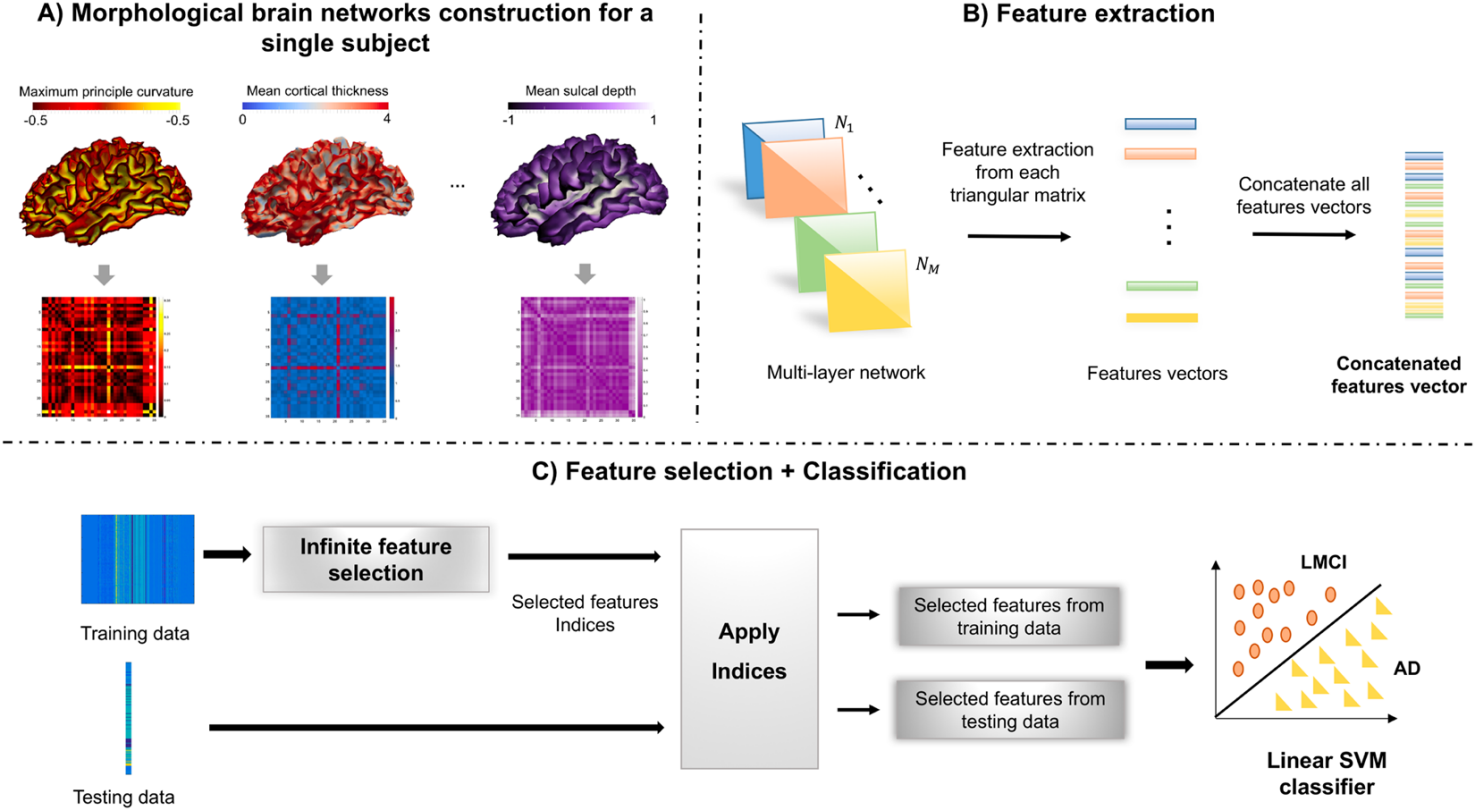
Illustration of AD/LMCI classification framework steps for the proposed cortical morphological network architectures. (**A**) We generate different morphological networks, each derived from a specific attribute of the cortical surface shape. (**B**) For each multi-layer network, we extract features from the triangular part of each cortical connectivity matrix. (**C**) For feature selection, we use IFS strategy (Roffo *et al*.^56^), then we train a linear support vector machine classifier using the selected connectional features.

We first introduce our morphological brain network construction strategy from structural T1-w MRI. Then, we propose two different architectures to explore the relationship between multiple brain connectivity morphological views: (1) a deep multi-level similarity network that aggregates different morphological brain networks with hierarchical combinations of similarity networks between them; and (2) morphological brain multiplex network, which is defined through inserting additional inter-layers between the aggregated networks. Last, we perform feature extraction and selection to classify a testing subject, and morphological biomarker identification. Figure 4 displays the key steps of the proposed framework.

### Morphological Brain Network Definition

Following the cortical surface parcellation into *R* anatomical regions, for each ROI *R*_*i*_, we average the cortical attribute *a* across all vertices *v* in *R*_*i*_ as follows:

$$\frac{1}{\#\{v\in {R}_{i}\}}\sum _{v\in {R}_{i}}a(v),$$

where #{*v*∈*R*_*i*_} denotes the number of vertices *v* belonging to ROI *R*_*i*_, and *a*(*v*) the cortical attribute value assigned to vertex *v*. Ultimately, to define the morphological connection *N*_*a*_(*i*,*j*) in network *N*_*a*_ between ROIs *R*_*i*_ and *R*_*j*_, we compute the absolute difference between averaged cortical attributes in both ROIs:

$${N}_{a}(i,j)=|\frac{1}{\#\{v\in {R}_{i}\}}\sum _{v\in {R}_{i}}a(v)-\frac{1}{\#\{v\in {R}_{j}\}}\sum _{v\in {R}_{j}}a(v)|.$$

Given *R* cortical regions in each hemisphere, the size of each fully connected morphological network is *R* × *R*. We note that according to our definition, as two ROIs *R*_*i*_ and *R*_*j*_ become more similar in morphology, *N*_*a*_(*i*, *j*) tends to 0.

### Proposed Morphological Network Architectures

To extract relevant and high-order morphological features from a set of *M* morphological cortical brain networks {*N*_1_, .., *N*_*M*_}, each encoding a specific shape attribute of the cortical surface, we propose ‘simple-to-complex’ strategies for building network architectures that capture different characteristics of how these networks interact with one another. In particular, high-order network architectures aim to reflect how these networks are nested with respect to one another in a high dimensional manifold of networks.

### Proposed deep similarity network architecture construction

**Figure 5:**
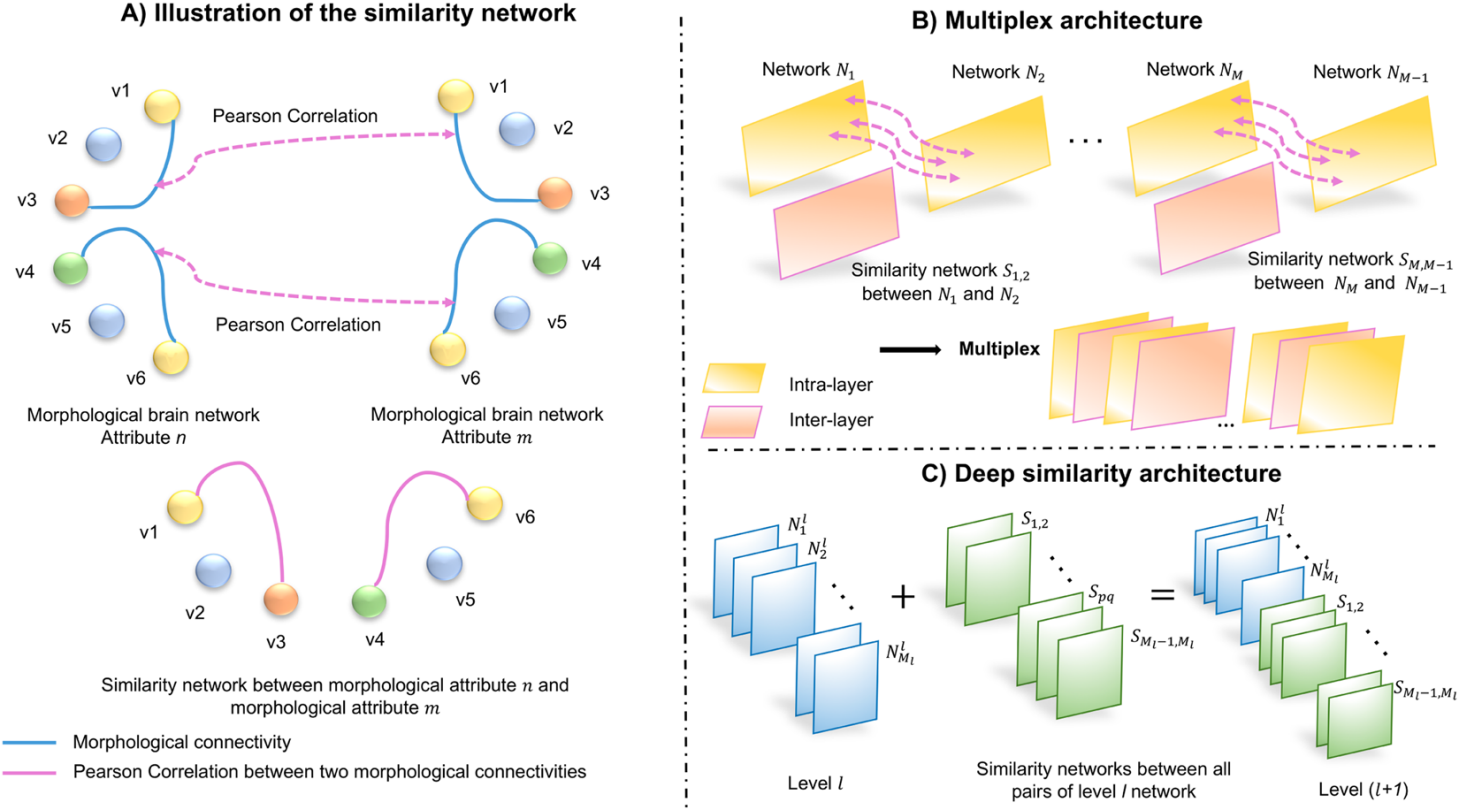
Proposed deep similarity network and multiplex network architectures, with illustration of the similarity network construction step. (**A**) We generate the similarity network between two morphological attribute networks by computing Pearson Correlation between them. (**B**) We construct the multiplex architecture where each inter-layer is a similarity network. (**C**) We consider the inter-relations between ${{\mathscr{N}}}^{l}$ networks through progressively concatenating, from a previous level *l* to a current level (*l* + 1), all possible similarity networks between pairs of networks.

We first propose a deep multi-level network architecture, where each level integrates the similarity networks between all pairs of networks in the previous level. The relationship between pairs of networks is defined by the measure of Pearson correlation (Fig. 5A). Thus, we define the degree of correlation between different cortical networks at each level. The baseline level (*l* = 0) is composed of all concatened networks ${{\mathscr{N}}}^{0}=\{{N}_{1}^{0},\mathrm{..},{N}_{M}^{0}\}.$ To build the next level, we create a larger multi-layer network through concatenating *n*_*s*_ similarity networks, where ${n}_{s}={C}_{M}^{2}=M!/(M-2)!2!$, representing the number of possible pairwise combinations between *M* networks. This produces a new deeper network ${{\mathscr{N}}}^{1}={{\mathscr{N}}}^{0}\cup \{{S}_{1,2},\ldots ,{S}_{pq},\ldots {S}_{M-1,M}\}$, where *p* and *q* represent the indices of two different networks in ${{\mathscr{N}}}^{0}$. For brevity, we note the baseline network at a specific level *l* as ${{\mathscr{N}}}^{l}=\{{N}_{1}^{l},\ldots ,{N}_{{M}_{l}}^{l}\}$, where *M*_*l*_ represents the total number of level *l* networks. Hence, in the next level (*l* + 1), we consider ${{\mathscr{N}}}^{l}$ as the baseline network, and add the similarity networks at a specific level *l* + 1 as: ${{\mathscr{S}}}^{l+1}=\{{S}_{1,2},\ldots ,{S}_{pq},\ldots {S}_{{M}_{l}-1,{M}_{l}}\}$, where *S*_*pq*_ represents the similarity network between networks ${N}_{p}^{l}$ and ${N}_{q}^{l}$. From level to level, we gradually add similarity networks between networks in the previous level (including similarity networks), thereby producing deeper networks from one level to the next one, where ${{\mathscr{N}}}^{l+1}={{\mathscr{N}}}^{l}\cup {{\mathscr{S}}}^{l+1}$ (Fig. 5C). The deep multi-level similarity network architecture is thus constructed in a hierarchical way, which captures not only network-to-network similarities, but also ‘similarity-to-similarity’ similarities.

### Proposed ensemble multiplex network architecture construction

Although the proposed deep similarity network architecture allows to explore similarities between networks at different hierarchical levels, this aggregates the similarity networks at the end of previous multi-layer network, in an agglomerative manner without enabling us to take account into the most correlated pairs of morphological networks. To enforce a more *structured* design of networks and their similarities, we propose to use a multiplex network to model the inter-relations between different layers. In a generic way, we define a brain multiplex ${\mathcal M}$ as a set of *M* intra-layers {*N*_1_, .., *N*_*M*_} (i.e., morphological networks), where between two consecutive layers *N*_*i*_ and *N*_*j*_, we slide an inter-layer *S*_*i*,*j*_. This yields to following multiplex architecture: ${\mathcal M} =\{{N}_{1},{S}_{1,2},{N}_{2},\ldots ,{N}_{i},{S}_{i,j},{N}_{j},\ldots ,{N}_{M}\}$ (Fig. 5B). Unlike the previous architecture (Fig. 5C), we note that for a specific multiplex, we are only allowed to explore similarities between *consecutive* layers. We also use Pearson Correlation to generate inter-layers as the deep similarity network architecture. Hence, to explore the inter-relationship between all possible combinations of layers for each subject, we generate *K* multiplexes through simply reordering the intra-layer networks while fixing the first intra-layer, thereby generating an *ensemble of multiplexes* ${\mathbb{M}}=\{{ {\mathcal M} }_{1},\ldots ,{ {\mathcal M} }_{K}\}$. Each of these multiplexes captures specific similarities between different kinds of morphological networks (e.g., sulcal depth network and cortical thickness network) that may not be present in another brain multiplex.

### Network feature extraction and selection for classification

For each of the proposed subject-specific network architectures in the previous section, we perform feature extraction, selection and LMCI/AD classification as follows.

#### Feature extraction

To explore the discriminative power of each region-to-region morphological connectivity in the cortex, we directly use the weights of edges in the morphological network as connectional brain features.

Since the constructed morphological connectivity matrix (or network *N*_*a*_) is symmetric (Fig. 4A), connectional features are extracted for each subject through concatenating the weights of all connectivities in each triangular matrix. Of note, for each network of size *R* × *R*, we extract a feature vector of size (*R*(*R* − 1)/2). For the deep similarity network and multiplex architectures, we extract features from each network in the architecture, then concatenate them all together into a high-dimensional feature vector. For a network architecture comprising *M*_*n*_ networks, the size of the final feature vector is *M*_*n*_ × (*R*(*R* − 1)/2).

#### Feature selection and classification

Due to the high-dimensionality of the extracted feature vectors and the small number of data samples, feature selection is a key step in classification tasks to both reduce the dimension of the training feature vectors and single out the most discriminative features. To this aim, we train a support vector machine (SVM) classifier using leave one-out cross-validation (LOO-CV) strategy. Given *P* subjects, we apply Infinite Feature Selection (IFS)^56^ in a supervised manner using the (*P* − 1) training subjects to select the top *K*_*f*_ features that significantly distinguish LMCI from AD patients. The most frequently selected features across different cross-validation schemes represent the morphological connectional biomarkers that allow to distinguish between AD and LMCI patients.

IFS method is a filter-based algorithm that aims to avoid over-fitting in a high-dimensional data by not considering irrelevant and/or redundant features. Compared with other feature selection methods, IFS has a compelling aspect that allows to efficiently identify reliable distinctive features for classification tasks. Most feature selection methods, which rank and select features, evaluate the importance of each feature individually, usually by neglecting potential interactions among the elements of the joint set. However, IFS performs joint ranking with selection and the score attributed for each feature in influenced by all other features. The idea is based on building a graph for the feature distribution, where the vertices denote the features and the edges represent the pairwise relationships among the feature distribution. Then, the algorithm ranks different morphological features by their importance and discriminative power. It evaluates the importance of a given feature while considering all the possible subsets of features. Given the output indices of the ranked features, we select the top *K*_*f*_ ranked features to train a linear SVM classifier using LOO-CV strategy to assign a label (LMCI or AD) to a new testing subject **(**Fig. 4C**)**.

#### Identification of morphological connectional biomarker

To identify morphological connectional biomarkers, we select the top *n*_*f*_ indices of *K*_*f*_ discriminative features ranked by IFS^56^ across all *P*LOO-CV. Specifically, we generate a matrix of size *n*_*f*_ × *P*, where each row represents the top ranked indices of features and each column represents a specific rank. For a given feature *f*_*k*_, we calculate its normalized rank across different LOO-CV as follows: $r({f}_{k})=(\sum _{i=1}^{P}\sum _{j=1}^{{n}_{f}}{\delta }_{ij}{w}_{ij}({f}_{k}))/P$, where *δ*_*ij*_ = 1 if *f*_*k*_ is selected, *δ*_*ij*_ = 0 otherwise. The weight *w*_*ij*_(*f*_*k*_) denotes the corresponding weight of feature *f*_*k*_ assigned by IFS, at the *i*^*th*^ LOO and *j*^*th*^ rank. Next, we identify the connectional biomarkers as features with the top normalized ranks.

### Availability of materials and data

The data that support the findings of this study are available from ADNI data (http://adni.loni.usc.edu/). For reproducibility and comparability, the authors will make available upon request all morphological networks generated based on the four cortical attributes (maximum principal curvature, cortical thickness, sulcal depth, and average curvature) for 77 subjects (41 AD and 36 LMCI) following the approval by ADNI Consortium. The Matlab code for generating an ensemble of multiplexes using *M* brain networks for a single subject (e.g., morphological, structural, or functional) is also available from the authors upon request.
